# Peer counselling as an approach to improve complementary feeding practices: a narrative review

**DOI:** 10.1186/s41043-023-00408-z

**Published:** 2023-07-04

**Authors:** Nabila Binte Haque, Seema Mihrshahi, Rukhsana Haider

**Affiliations:** 1 Department of Health Systems and Populations, Faculty of Medicine, Health and Human Sciences, Macquarie University, Sydney, NSW 2109 Australia; 2 Health and Nutrition (TAHN) Foundation, Banani, Dhaka 1213 Bangladesh

**Keywords:** Complementary feeding practices, Peer counselling, Infant and young child feeding

## Abstract

**Background:**

Appropriate complementary feeding can help reduce the risk of malnutrition and is especially important in Asian and African countries. Peer counselling has been used as an approach to improve complementary feeding practices and is often combined with other interventions, like food fortification or supplements, or as a part of broader nutrition education program. The aim of this narrative review is to assess the effectiveness of peer counselling on improving complementary feeding practices in Asian and African countries.

**Methods:**

We searched through seven electronic databases: CINAHL, MEDLINE (OVID), PubMed, Embase, Web of Science, the Cochrane Library and WHO Global Health library from 2000 to April 2021, and had the following inclusion criteria. Studies were included if they were community- or hospital-based, had infants aged 5–24 months old, had individual or group peer counselling, and the effects of peer counselling on complementary feeding practices were measured. Methodological quality was assessed using the Joanna Briggs Institute’s critical appraisal checklist for evidence studies.

**Results:**

Out of 6 studies that met the above criteria, 3 studies were randomised controlled trials and 3 were quasi-experimental studies. In Bangladesh, India, Nepal and Somalia, peer counselling was found to be effective in improving timely initiation of complementary feeding, minimum meal frequency and minimum dietary diversity in all of our selected studies. In addition, improvement in breastfeeding practices, complementary foods preparation, hygiene, psychological stimulation for cognitive development of children and mothers’ understanding of hunger cues were observed in some of our selected studies.

**Conclusions:**

This review evaluates the effectiveness of peer counselling to improve complementary feeding practices in Asian and African countries. Peer counselling improves timely complementary feeding and ensures the correct proportions and consistency of foods including adequate amounts of food is given. Other important complementary feeding indicators like minimum dietary diversity, minimum meal frequency and minimum acceptable diet can also be increased through peer-counselling interventions. Peer counselling is well known to enhance the rate of breastfeeding practices, but this review suggests it is also effective for complementary feeding and may inform future nutrition programs to extend the length of peer counselling for mothers.

## Background

Malnutrition has become a pressing global concern and is one of the leading causes of morbidity and mortality in children [1]. Malnutrition alone is responsible for almost 45% of child mortality globally [2]. Sub-Saharan Africa and South Asia have the highest child mortality rates in the world, and 80% of child mortality refer to the sub-Saharan and South Asia regions due to birth complications and undernutrition [2, 3]. Inadequate complementary feeding practices are a major cause for the onset of malnutrition [4]. Appropriate complementary feeding can help reduce malnutrition and other associated diseases [5, 6].

Complementary feeding is defined as ‘the consumption of foods and liquids when breast milk is no longer sufficient to meet the nutritional requirements of infants’ [7, 8]. The World Health Organization (WHO) recommends the age of 6 months as appropriate for ‘timely initiation of complementary feeding’ practices for infants. This means an infant should be fed solid, semisolid or soft complementary foods in addition to breastmilk for up to 2 years [5, 8]. Focusing on improving feeding practices of children under 2 years is critical because they experience rapid growth and development and a higher vulnerability to illness during this time [4]. Poor complementary feeding practices leads to many illness such as diarrheal diseases, measles, malaria and respiratory infections [9].

Many studies have been conducted to improve complementary feeding in infants. Preventive interventions, such as nutrition education, using fortified food powder or other supplementation, food vouchers or ready to use therapeutic foods, have been trialled [10, 11].

Positive impacts of feeding counselling on improving energy and nutrient intake of children under 2, as well as improving their growth, are ever-increasing. Counselling to improve breastfeeding practices among mothers has proven to be quite successful in the past [12]. Nutrition counselling, without any other supplementation, has been shown to improve nutritional status of a child (from mild underweight to normal weight) by 37% after just 3 months of intervention [13].

The term ‘counselling’ is often used in infant and young child feeding (IYCF) studies. Counselling is a type of support or help provided to mothers, fathers or sometimes other family or household members [14]. Positive effects of counselling on people’s behaviours and knowledge is widely acknowledged and accepted [15]. Counselling can be provided through various mediums such as health professionals, community workers, women’s groups, midwives, nutrition professionals, mothers and fathers groups or peer counsellors [16].

Peer counselling is a support process that involves a one-on-one interaction or interaction between members of a group, where the peer counsellors provide advice and teach skills to each other within group members. Various studies have shown that peer support can help improve many aspects of health [17, 18]. Although the effects of peer counselling on breastfeeding have been well known, the effectiveness of peer counselling or peer support as an intervention for influencing complementary feeding in infants is less known, and very few studies have been designed to study this aspect [12, 19–21].

This paper is narrowly focused on a particular topic with a few selected peer-reviewed papers that provided an overall interpretation of peer counselling in a critical and comprehensive way as a form of narrative review. The aim of this narrative review is to analyse the evidence on published peer-supported studies that specifically includes peer-counselling and peer supported interventions and seeks to improve complementary feeding practices in Asian and African countries.

## Methods of literature searching

### Search strategy

We searched 7 electronic databases: CINAHL, MEDLINE (Ovid), PubMed, Embase, Web of Science, the Cochrane Library and WHO Global Health library, using the keywords ‘peer counselling’ AND ‘complementary feeding’ AND ‘intervention’ [the search strategy for MEDLINE (OVID) is presented as an example in Appendix Table 4. The same strategy was used for other databases for studies published in English and with publication data from 2000 to April 2021. Titles and abstracts were initially screened by review author (NBH) and a research librarian (JC). Potential full texts to be included were screened separately between the 3 authors (NBH, SM, RH) who confirmed each study against the agreed exclusion and inclusion criteria. Disagreement was resolved by discussion amongst the authors (NBH, SM, RH).

### Study selection and eligibility criteria

A study was eligible for inclusion if it:

- Was a randomised or non-randomised controlled trial (a randomised trial study had a control group and a non-randomised study may have a control group or may measure the effects based on pre- and post-intervention results).
- Consisted of mother–infant pair participants.
- Had infants who were aged from 5–24 months old during the intervention phase (5 months to see the outcomes of early initiation)
- Included some components of individual or group peer support or peer counselling (as defined below) as the intervention
- Related the study outcomes to complementary feeding practices (can be either primary or secondary outcome).

To be included as a peer-counselling study, studies could be:

- Community-based or hospital-based
- Individual or group counselling, with peer counsellors from the same geographic region or residing in the same community
- Peer counsellors from the same level of education as the target group

males or females who are solely focused on peer counselling and no other occupation (e.g., community nurse, community health workers, nutritionists).

Interventions that have counselling provided by health professionals, healthcare providers, midwives, nutrition counsellor or others, without involvement of any peer support (as defined above), were excluded from the study. Studies with other types of interventions, such as food supplementation, food fortification, ready to use therapeutic food, nutrition education or behaviour-change models, were also excluded in the same way if they had no form of peer support as part of the intervention. We excluded pilot studies, descriptive or observational studies or grey literatures as well.

### Data extraction

Two of the review authors (NBH and SM) independently extracted data in a specific tabulated form, which included country of study, study type, study design, study year, targeted population, objectives, sampling technique, eligibility criteria, intervention methods, outcomes, data collection methods and analysis procedures. Outcomes were measured as per the complementary feeding measurement indicators set by World Health Organization (WHO) [22, 23] (Appendix Table 5). This included timing of initiation of complementary feeding, minimum meal frequency (MMF), minimum dietary diversity (MDD) and minimum acceptable diet (MAD). Other relevant measures, such as use of separate cooking pots, using extra oil in baby’s food, and hygiene during food preparation, were also included.

### Quality of evidence assessment

Two of the review authors (NBH and SM) independently assessed the methodological quality of selected studies using critical appraisal checklist [24] by the Joanna Briggs Institute (JBI) manual for RCTs [25] and quasi-experimental studies [26]. The strengths and limitations were presented in a tabular form (Appendix Tables 6 and 7) for all studies. Studies were categorised into good and moderate quality studies based on their overall appraisal inclusion and exclusion criteria. If the selected studies met all included criteria in the JBI appraisal checklist, they were scored as good quality. Otherwise, the studies scored as moderate quality studies if they failed to meet some inclusion criteria from the checklist. Any discrepancies were resolved through discussion with the review team (NBH, SM, RH).

## Results

### Search results

**Fig. 1:**
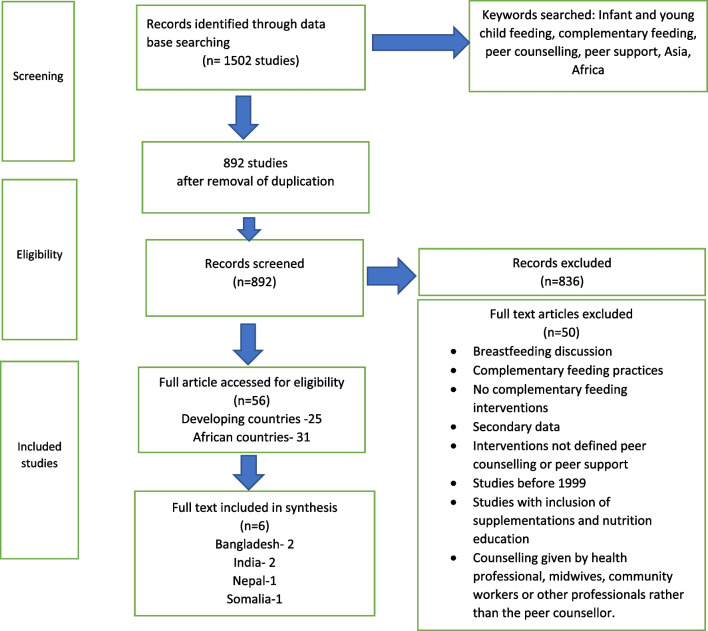
Flow diagram of included and excluded studies

A total of 1502 original records were found during database searches; out of these, 610 studies were duplicates and were subsequently removed. After screening the titles and abstracts of the remaining 892 studies, 56 studies were selected for further consideration. After full-text screening, 50 articles were excluded due to ineligible study populations, mixed type of intervention, studies not considered as peer-counselling intervention, outcomes not related to complementary feeding and ineligible study design. The study selection and screening process is presented in a flow chart in Fig. 1.

Only 6 studies met the inclusion criteria outlined above. Among the selected studies, 2 were conducted in Bangladesh [27, 28], 2 in India [29, 30], 1 in Nepal [31] and 1 study in Somalia [32]. Characteristics of the included studies are given in Table 1.

### Intervention

The type of intervention and the delivery provider, the number, duration, frequency, intensity of session in the interventions, varied between studies. A summary of the intervention characteristics is presented in Table 1.

**Table 1:** Characteristics of selected studies (*n* = 6)

Studies, author and year	Location/Area	Targeted population	Study Design	Intervention group and interventions	Comparison group	Duration of the interventions
Ara et al. 2019 [28]	Mirpur slums, Dhaka, Bangladesh	Mother–infant pairs, A. married pregnant Women aged 16–49 years, their in-laws’ members and fathers, B. child aged 6–12 months	Community-based cluster Randomised controlled trial	IYCF counselling, peer counselling with psychosocial stimulation, feeding bowl and spoon, handwashing solution, and homemade toys	Usual health and nutrition messages by the other facilities	January 2015- June 2016, 1 year, started from birth to 12 months of child’s age
Aboud et al. 2011 [27]	Khansama subdistrict, Northwest Rural, Bangladesh	Mother–infant pair, Children aged 8–20 months	Cluster‐randomised field trial	1st IG: 18 Informational sessions on health and nutrition by peer educators (RFS) 2nd IG: 18 Sessions, food powder fortified with minerals and vitamins (RFS + +)	CG: Regular 12 informational sessions on health and nutrition by local community health workers provided from the program as usual care	April–December 2008, Outcomes measured after the end of 2 weeks and 5 months of intervention
Vandana et al 2014 [30]	Urban slum, Delhi, India	Mother–infant pair, A. mothers from 5th month of postpartum B. children of 6–12 months age	Randomised controlled trial	IG divided into 3 subgroups; 1st IG (A)- counselling by a nutrition professional 2nd IG (B1) and 3rd IG (B2)- counselling by peer counsellors who were local health workers	CG: No counselling	No specific month/year found, intervention started from 6th month of pregnancy and ends 12 months of child’s age
Kushwaha KP et al. 2014 [29]	Lalitpur, Uttar Pradesh, India	Mother–infant pairs, Mothers who had delivered a child within 0–3, 3–6, 6–12, and 12–24-months age	Quasi-experimental (pre-intervention- T0, Intervention phase 2 years- T1, post-intervention phase 5 years—T2)	IG- Peer counselling by mother support group (MSG), home visits, surveys	No CG, Outcomes measured at baseline in 2006 and compared with pre-intervention 2008(T1) and 2011(T2) post-intervention	Dec 2006 to 2011
Abdullahi et al. 2019 [32]	Rural Galgadud and Bari region, Somalia	Pairs including Mother–children’s pair and father–children’s pair, children aged 6–12 months	Quasi-experimental	IG- Intervention was peer counselling based on 3 groups; 1st IG- Kismayo (who had both F2F & M2M) 2nd IG-Adado (Only M2M) 3rd IG-Armo (Only F2F)	CG- No counselling, (For MDD, CG was based on 2 groups and same intervention as IG: 1st CG-Adado (Only M2M group): 2nd CG- Armo (Only F2F group)	Aug 2018 to Dec 2019
Singh et al 2018 [31]	Rural Bhojpur, Bajhang and Rupandehi. Nepal	Mother–infant pair in end line, Children aged 6–23.9 months	Quasi-experimental	IG- Peer facilitation or mobilisation	CG- No counselling,	2011 to 2016

*BF* Breastfeeding, *BMI* Body mass index, *CG* Control group, *CF* Complementary feeding, *F2F* Father-to-father group, *IG* Intervention group, *IYCF* Infant and young child feeding, *MDD* Minimum dietary diversity, *MMF* Minimum meal frequency, *MAD* Minimum acceptable diet, *M2M* Mother-to-mother group, *MSG* Mother support group, *PC* + *PCS* Peer counselling plus psychological stimulation, *RFS* Responsive feeding and stimulation, *RFS* + Responsive feeding and stimulation plus sprinkles, *RCT* Randomised controlled trial *SAM* Severe acute malnutrition, *SD* Standard deviation, *T0* Pre-intervention phase, *T1* Intervention phase, *T2* Post-intervention phase

### Intervention type and provider

In 5 out of 6 studies, interventions were delivered by community-based peer counsellors [27–29, 31, 32], and in the other study the intervention was provided by a dual approach, where the intervention was delivered using both community health workers and a nutrition professional who fulfilled the definition of peer counselling as described above [30].

In 5 of our selected studies [27, 28, 30–32], the sessions were delivered face-to-face via either individual home visits or group meetings. In 1 quasi-experimental study, counselling was delivered using both home visits and later in a hospital [29].

### Intervention numbers, frequency and sessions

Selected studies varied in intensity as some provided one-to-one counselling, while others provided group counselling [27–32]. Intervention visits ranged between 2 and 10 times in the first 6 months of a child’s age. One study in Bangladesh made 13 regular visits: 2 were before and during the first month of delivery, 7 visits occurred when the child was between 2 and 8 months and then single monthly visits during the child’s 10th and 11th month of life [28]. In the Delhi study, counselling was delivered in 3 visits during the 7th, 8th and 9th month of pregnancy and 2 further sessions were conducted when the baby was 5 months old [30]. The Lalitpur study provided intensive nutrition peer counselling, which was divided into 3 phases: 10 visits in first 6 months of the baby’s life (T1 phase), 6 visits during the next 6 months of life and then 3 follow-up visits in the 2nd year of the intervention phase (T2 phase), when the child was between 12 and 24 months [29].

Another study in Bangladesh was divided into 2 intervention groups and 1 control group for children aged 8–20 months [27]. Both intervention groups and the control group received 12 counselling sessions [27]. In addition, the 1st intervention group received 6 extra intensive nutrition counselling sessions and the 2nd intervention group received extra food sprinkles with 6 extra intensive counselling along with regular counselling [27]. Unfortunately, the study in Nepal lacked relevant information about the number and type of counselling sessions [32]. The study from Somalia provided 12 regular IYCF sessions for children aged 6–12 months for over a 3-month period [32].

### Peer counsellors

#### Socio-demographic characteristics of peer counsellors

Although the age groups of the peer counsellors varied from young to middle age, the actual ages of the counsellors were not mentioned in most of the studies [27–32]. The majority of the peer counsellors were female [27–31], except in the study from Somalia, which included both male and female peer counsellors [32].

Local community health workers worked as peer counsellors in 2 of the studies [27, 30]. In 3 of the selected studies, married women with breastfeeding experience or at least 1 child under 2 years of age were selected as peer counsellors [28, 30, 31]. An Indian study used mother support groups (MSGs) of 3–4 members, including traditional birth attendants, experienced mothers, and community or nutrition workers, as their peer counsellor [29].

#### Recruitment and workload

Peer counsellors were all recruited from the same community, as specified in the inclusion criteria [27–32]. The number of peer counsellors allocated to intervention participants varied from one study to another; therefore, their workload also varied (Table 2).

**Table 2:** Peer selection and training of selected studies (*n* = 6)

Study author, year	Type of peers	Number of peers	Training details	Training given by	Training duration
Ara et al. 2019 [28]	Women with BF experience, community-based	10	The WHO/UNICEF breastfeeding counselling course, topics covered listening and understanding mothers’ difficulties, position and attachment during breastfeeding, communication skills, give support to mothers, providing relevant help, practical help and others Training taught by demonstration, role play	No information	40 h training duration (4 h daily for 10 days)
Aboud et al. 2011 (24)	Local young women who worked as community health worker, Community-based	No information	Informational sessions on health, nutrition and child development, topics included responsive feeding, self-feeding, dietary diversity, hygiene, behavioural strategy Training taught by practically, verbally and using flip charts	No information	4 days training, 30-page manual
Vandana et al 2014 [30]	Local health workers; Community-based	2	Trained for promoting optimal infant feeding practices, no further details of training	Nutrition professional	No information
Kushwaha et al 2014 [29]	Mother support groups (experienced mothers, traditional birth attendant, community health/nutrition worker), both facility- and community-based	48	International Baby Food Action Network (IBFAN/WHO/UNICEF/Breastfeeding promotion network of India-BPNI), topics were an integrated course on breastfeeding, complementary feeding, infant feeding and HIV Training taught information unavailable	Project stuffs trained the facility trainers and later facility trainers trained others	7 days training, 3 in 1 module
Singh et al 2018 [31]	Mothers with BF experience, Community-based	16	Training on key MIYCN messages, topics were health and nutrition, antenatal and postnatal care, pregnancy and lactation period knowledge including iron folic acid intake, extra meal, etc., taught practically providing visual aids, picture books	Project and health facility staffs	4 days training, 3 modules in 4 sessions
Abdullahi et al. 2019 [32]	Mothers and fathers, Community-based	11 [5 lead mother and 6 lead father]	Training on child nutrition and diseases specific issues**,** using their existing training modules, No further details found	No information	5 days’ workshop, 12 Infant and young child feeding (IYCF) sessions

*BF* Breastfeeding, *CF* Complementary feeding, *MIYCN* Maternal infant and young child nutrition

One study from Bangladesh recruited 10 peer counsellors who served 350 mother–infant pairs, which included 30–35 mothers in each of the clusters [28]. While the Lalitpur study recruited 48 peer counsellors, who served 1426 mother–infant pairs at 2 time points: T1 phase (after 2 years of intervention) and T2 phase (after 5 years of intervention) [29].

Both 2 peer counsellors delivered the intervention to 426 mother–infant dyads in the Delhi study, where 213 were pregnant women and 198 were mother–infant dyads of 6–12 months [30]. The study in Nepal had 16 peer counsellors who served 1890 mother–infant pairs using baseline (April–May 2014) and end-line (May–June 2015) data [31].

Another study in Bangladesh provided no clear information about the number of peer counsellors serving 302 mother–infant pairs [27]. The study in Somalia, which used baseline and end-line surveys, reported 18 peer counsellors, including male and female counsellors, who served 250 mother–infant pairs [32]. They had 9 mothers and 9 fathers as peer counsellors who were responsible for providing the counselling to 10 mothers and 10 fathers within their own communities [32].

#### Training

All studies reported some form of training for their peer counsellors [27–32]. Training durations and sessions varied from one study to another between 4 and 10 days apart from 1 study, which did not mention the time duration of the training [30]. The main messages conveyed from the training related to infant and young child feeding (IYCF) and study objectives. Three studies provided additional training materials such as picture books, flip-charts and handbooks [27, 30, 31]. Two of the selected studies provided follow-up support with monitoring and supervision of peer counsellor’s performance [28, 29]. Details of the peer counsellor trainings are described in Table 2.

### Key findings related to complementary feeding

The key findings from each of these studies are described below and are summarised in Table 3.

**Table 3:** Summary of selected studies complementary feeding outcomes (*n* = 6)

Author and year	Sample size (mother–infant pairs)	Outcome measures on CF	Reported outcomes				
Ara et al. 2019 [28]	350		Odds ratio (OR)			95% CI	
		MDD (7–12 months)	1.98			1.37, 2.87	
		MMF (7–12 months)	1.03			0.60, 1.76	
		MAD (7–12 months)	1.30			0.85, 2.01	
		Flesh foods	1.95			1.38, 2.77	
		Eggs	1.42			1.00, 2.77	
Vandana et al. 2014 [30]	426, (213 pregnant women, 198 mother–infant dyads of 6–12 months)	Initiation of CF	Intervention group (IG)			Control group (CG)	
			A (%)	B1 (%)	B2(%)	CG	
		< 6 months	1.9	4.0	2.0	48.9%	
		6–7 months	76.4	74.0	78.0	19.4%	
		> 6 months	21.5	24.0	20.0	25.2%	
		7–12 months	Universal coverage			93.6%	
		MDD (At 6–12 months) (mean consumption)	37.1%			6.4%	
Singh et al. 2018 [31]	1890 (end-line survey in 3 districts)	MDD (At 7–23.9 months) (Mean consumption)	Intervention group			Control group	
			Baseline	End-line		Baseline	End-line
		0–7 food groups	3.1%,	3.5%		3.2%	3.4%
		≥ 4 food groups	38.9%	45.8%		32.7%	48.7%
Aboud et al. 2011 [27]	302	MDD (Based on 0–7 food groups) Mean consumption	Intervention group			Control group	
			3.07			2.96	
Abdullahi et al. 2019 [32]	250	MDD (Based on 7 food groups) (Mean consumption)	Intervention group			Control group	
		Baseline	11%			12%	
		End-line	16%			14%	
Kushwaha KP et al. 2014 [29]	1426 [425 (T0), 480 (T1) and 521 (T2)	Timely initiation of CF (at 6–8 months)	Intervention phases (intervention, T1 and post-intervention, T2)			Control phase (pre-intervention, T0)	
			T1 (OR: 5.6, 95% CI: 3.6, 8.7) 85%, T2 (OR: 22.9, 95% CI: 11.8, 44.1) 96%			T0 54%	
		CF along with BF (up to 2 years)	T1 (OR: 6, 95% CI: 1.15, 31.4) 36%, T2 (OR: 8.06, 95% CI: 1.96, 49.1) 42%			T0 4.5%	

*adjOR* Adjusted odds ratio, BF- breastfeeding, *CF* Complementary feeding, *CG* Control group, *CI* Confidence interval, *IG* Intervention group, *MMF* Minimum meal frequency, *MDD* Minimum dietary diversity, *MAD* Minimum acceptable diet

### Peer-counselling effects on timely initiation of complementary feeding

Out of the 6 studies, peer counselling was found to have improved timely introduction of complementary feeding in 2 studies [29, 30]. Four studies had no measurement for introduction of complementary foods [27, 30–32]. Both studies reported that the time of starting complementary feeding of target groups was, on average, 6–8 months of age [29, 30].

The Delhi study divided introduction of complementary foods according to age groups [30]; 78% of the infants from the intervention groups started receiving solid or semisolid foods when they were between 6–7 months of age [30], whereas only 19.4% children from the control group received solid or semi-solid foods at 6–7 months [30].

The Lalitpur study reported a significant effect (*P* = 0.001) of peer counselling on the timely initiation of complementary foods in both of the intervention phases—T1: 2 years post-intervention phase (OR 5.6, 95% CI 3.6, 8.7) and T2: 5 years post-intervention phase (OR 22.9, 95% CI 11.8, 44.1) compared to T0: pre-intervention phase [29].

### Peer-counselling effects on minimum meal frequency (MMF)

Only 1 study in Bangladesh (the study by Ara and colleagues) [28] measured minimum meal frequency and reported increased meal frequency in the intervention group compared to the control (adjOR 2.08, 95% CI 1.39, 13.11, *P* = 0.001) [28].

### Peer counselling effects on minimum dietary diversity (MDD)

Of the 6 studies, 5 measured dietary diversity [27, 28, 30–32], though measurements were taken in different ways. In most of the studies, MDD was measured based on the recommended 7 food groups. One study measured MDD based on cereal intake and another study measured it based on protein intake only. All 5 studies showed significant effects of peer counselling in improving child’s dietary diversity [27, 28, 30–32].

One study in Bangladesh assessed minimum dietary diversity based on protein (egg, flesh, organs) consumption [28]. Children aged between 7–12 months in the intervention group had almost twice as high (1.95 times) minimum dietary diversity (MDD) compared to the control group [28]. Egg (*P* = 0.045), fleshy food (*P* = 0.001) and overall total protein consumption (*P* = 0.001) in the intervention group were also significantly higher [28].

The study in Nepal measured MDD when the child was between 7–23.9 months old based on the 7 food groups—most of the children from the intervention group received at least 4 out of the 7 food groups, which was much higher than the children from the control group [31]. Mean difference of MDD was 4 times higher in the intervention group compared to the control group [31]. Both intervention groups (38.9% to 45.8%) reported improved dietary diversity at the end-line [31].

The study based in Delhi showed that the rate of attained MDD in the intervention group was 37.1%, as a direct result of peer counselling, compared to only 6.4% in the control group [30]. This study also mentioned peer counsellors’ effect on improving complementary food preparation methods, feeding skills and hygiene practices among the mothers [30].

In another study from Bangladesh, most of the children from the intervention group received food from at least 4 or more food groups out of the 7. At the end of the study, the intervention group’s mean MDD increased compared to the control group [27].

The study in Somalia categorised the food diversity in two parts: low dietary intake (less than 3 food groups out of the 7) and high dietary intake [4 or more groups out of the 7] [32]. At the end of the study, the intervention group reported higher dietary intake compared to the control group [32].

#### Peer-counselling effects on minimum acceptable diet (MAD)

Only one study from Bangladesh measured the effectiveness of the peer counselling on minimum acceptable diet, and the results were not significantly different between the control and intervention groups [28].

#### Quality assessment of the selected studies

The majority of studies (*n* = 4) were categorised as good quality [29–32]; 2 were categorised as moderate quality studies [27, 28] according to our quality assessment [25, 26]. In the studies categorised at moderate quality, it was unclear whether they met the requirement for follow-up participants, outcome measurement and statistical analysis [27, 28]. No studies were excluded on the basis of their study quality.

#### Breastfeeding and other outcomes

Out of the 6 studies, 5 had breastfeeding outcomes relating to the effects of peer counselling [28–32]. The Lalitpur study, India [29], the study in Bangladesh [28], the study in Delhi, India [30], the study in Somalia [32] and the study in Nepal [31], all reported increased rates of early initiation of breastfeeding, or colostrum feeding, followed by exclusive breastfeeding as a result of the peer counselling. Breastfeeding and other outcomes such as child nutritional status, child development, responsiveness, anthropometric measures (e.g., height, weight and length gain) and infant and young child feeding (IYCF) knowledge are listed in Appendix Tables 8 and 9.

## Discussion

To the best of our knowledge, this is the first narrative review that focuses on studies designed to improve complementary feeding through peer counselling in Asian and African countries. We have identified 3 randomised controlled trials [27, 28, 30] and 3 quasi-experimental studies [29, 32, 33]. The findings of our study provide some evidence on the effectiveness of peer counselling in improving a child’s complementary feeding practices. Improvements in minimum dietary diversity (MDD) and minimum meal frequency (MMF) were reported in 5 out of the 6 studies, and all of them showed improvement in increasing MMF and MDD [27, 28, 30–32]. Timely introduction of baby’s 1st complementary food was improved in 2 studies, [29, 30], whilst the other 4 studies lacked information regarding timely introduction of complementary foods [27, 30–32]. We also found that 5 of the selected studies lacked enough information to address the effectiveness of peer support on minimum acceptable diet (MAD) [27, 29–32]. Similar result found in a community-based cross-sectional study in Ethiopia that investigated MAD and associated factors [34]. The study reported low prevalence rate of MAD (31.6%) due to the lack of promotion of IYCF practices in that community [34].

Our findings are consistent with previous evidence. A recent systematic review and meta-analysis compared the effects of community, financial and technology-based nutrition interventions by community health workers, mother support groups or peer counsellors [35]. The research included 83 studies from low- and middle-income countries [35]. The meta-analysis showed improvement in minimum dietary diversity (OR 2.34; 95% CI 1.17, 4.70) and minimum meal frequency (OR 2.31; 95% CI 1.61, 3.31) in children from the mother–peer group compared to those from the usual care group. The review also showed improved rates of early initiation of breastfeeding (EIBF), exclusive breastfeeding (EBF) and reduced wasting [35]. A scoping review focused on the behavioural-change interventions for complementary feeding in low- and middle-income countries in 64 studies, which explored complementary feeding [36]. The results showed that counselling (individual or group) was the most effective and commonly used platform to improve complementary feeding practices compared to any other social behaviour-change intervention [36]. A systematic review of qualitative studies explored views and experiences of women, peer supporters and health professionals in breastfeeding peer support studies and reported a better mutual understanding between the women group and the peer supporters [37]. The women groups also reported the positive influence of peer supporters that helped them to gain more confidence and encouraged them to continue the breastfeeding [37].

In this review, 5 studies identified improvements in breastfeeding along with complementary feeding [27, 29–32], and they all showed significant positive impacts of peer counselling in increasing early initiation of breastfeeding and exclusive breastfeeding [27, 29–32]. Positive results were also found in breastfeeding counselling to improve breastfeeding practices from many previous studies [12, 28–30, 38, 19].

Our selected 6 studies have reported outcomes that are related to behaviour change. Child development measures like height, length, weight along with overall nutritional status of a child also showed improvement. Peer counselling had positive impacts on social developments such as language capacity, social activities, encouraging child to self-feed as well as hygiene-related outcomes like establishing hand washing stations [27–32]. This complexity of interventions and outcomes makes it difficult to determine the precise effects of peer counselling as an individual intervention in order to improve complementary feeding outcomes.

We found that information on training methods and content of peer-counselling sessions in some of the studies were not sufficient. There was also no analysis on the cost effectiveness of the interventions. Overall, a large knowledge gap was absorbed by all the participants [27–32].

In 5 of the 6 studies, counselling was delivered at home via individual or group visits and the peer counsellors were local community members. Only the Lalitpur study used a community facility and later a hospital facility as intervention sites [29]. It is important to mention that one study (the study by Vandana et al. in Delhi) used professional nutrition counselling for 1 of the subgroups together with community health workers [30], who were only dedicated to peer counselling, and thus included in our study [30].

### Strengths

One of the strengths in this review is that studies were included only if peer counselling was conducted by local counsellors and the peer counsellors were responsible for counselling targeted complementary feeding only and nothing else, nothing else means no other responsibilities other than counselling. Being locals, it was easier for the peer counsellors to gain the mother’s trust and confidence. In similar studies, peer counsellors are also burdened with multiple responsibilities such as nutrition education for women and other supplementation for children. This may have contributed towards the success of the interventions and a more effective counselling process than any other studies like cross-sectional or cohort studies. All of the selected studies quality were generally good, but the majority of RCT studies did not have allocation concealment and were not able to blind participants to the study groups as they were receiving the intervention. The majority of quasi-experimental studies used non-uniform comparison groups, which may have affected the generalisation of the outcomes.

### Limitations

One of the main limitations was the small number of studies (only 6 studies) that met the inclusion criteria. We excluded a large number of studies because, although they had either nutrition counselling or nutrition education, they did not have peer counsellors. We also excluded grey literatures due to full text unavailability sometimes. Therefore, all of those restricted the number of studies.

Although many studies included counselling, not all could be considered as ‘peer counselling’. The majority of the studies used additional interventions with peer counselling, and the outcomes were measured in different ways. So, it was difficult to compare the studies with regard to interventions and outcomes. For example, 1 of the studies from Bangladesh used psychological stimulation together with peer counselling [28], whereas the other Bangladeshi study used fortified food powder [27]. Apart from the selected studies, we can also see other studies such as the study by Campbell and colleagues [20], which used chickpea, plumpy’doz and wheat soya blend++ as supplementary foods, in addition to child feeding counselling [20].

## Conclusion

This review highlights the positive effects of peer counselling on complementary feeding timing, minimum meal frequency, dietary diversity, all of which are significant for a child’s health and nutrition. This study focused solely on peer counselling, which can be used as an effective intervention in many low- and middle-income countries. This approach, if proven successful, can be adapted to include other nutrition actions and may inform other programs in the wider health sector.

## Data Availability

Not applicable.

## Appendix

See Tables 4, 5, 6, 7, 8 and 9.

**Table 4:** Keywords for search

Search	Search terms
S1	((“infant and young child feeding” OR “infant feeding” OR “feeding intervention”) [mp: title, original title, abstract, name of substance word, subject heading word, unique Identifier]
S2	((“complementary feeding” OR “complementary feeding interventions”) [mp: title, original title, abstract, name of substance word, subject heading word, unique identifier]
S3	((“peer counselling” OR “peer support” OR “peer groups” OR “counselling” OR “support” OR “Asia” OR “Africa”) [mp: title, original title, abstract, name of substance word, subject heading word, unique identifier]
S4	((“nutrition counselling” OR “nutrition education” OR “behaviour change intervention” OR “counselling”) [mp: title, original title, abstract, name of substance word, subject heading word, unique identifier]
S5	S2, S3 and S4
S6	limit S5 to (“infant and child < 6 months to 18 months > ” or “year = ”1999 to current” or “counselling < peer counselling > ” and English language)

**Table 5:** Definitions of complementary feeding indicators according to WHO and UNICEF. [22, 23] *Source*: WHO/UNICEF

Indicators	Description	Measurement
Initiation of complementary feeding	Introduction of complementary foods is the key indicator to understand whether complementary feeding started early, timely or delayed	It is measured as the percentage of infants at age 6–8 months who consumed solid, semisolid or soft foods
Minimum meal frequency (MMF)	The minimum meal frequency captures the caloric sufficiency of a child’s diet.’	It is measured as the minimum number of times, or more, as following;
		Minimum solid, semisolid, or soft foods (including formula) for non-breastfed children: 4–5 times at 6–23 months
		Minimum solid, semisolid, or soft foods for breastfed children: 2–3 times at 6–8 months and 4 times at 9–23 month with additional snacks 1–2 times per day
Minimum dietary diversity (MDD)	The minimum dietary diversity is the proxy indicator of mean micronutrient density adequacy of a child’s diet and it is measured by counting the numbers of food groups.’	Children aged 6–23 months should consume 5 out of 8 recommended food groups. The recommended 8 food and beverages groups are: 1. breast milk 2. grains, roots, and tubers 3. legumes and nuts; 4. dairy products (milk, yogurt, cheese, infant formula) 5. flesh foods (meat, fish, poultry and liver/ organ meats); 6. eggs; 7. vitamin-A rich fruits and vegetables, and 8. other fruits and vegetables
Minimum acceptable diet (MAD)	‘The minimum acceptable diet indicator captures a child’s diet as a proxy of energy adequacy and micronutrient density which measures the proportion with different requirements for breastfed and non-breastfeed child.’	This indicator combines minimum meal frequency and minimum dietary diversity

**Table 6:** Joanna Briggs Institute’s critical appraisal checklist of randomised controlled trials for study quality assessment**.** [25] *Source*: Joanna Briggs Institute’s critical appraisal checklist

Selected RCT studies strengths and limitations (*n* = 3)													
Studies	Was true randomisation used for assignment of participants to treatment groups	Was allocation to treatment groups concealed	Were treatment groups similar at the baseline	Were participants blind to treatment assignment	Were those delivering treatment blind to treatment assignment	Were outcomes assessors blind to treatment assignment	Were treatment groups treated identically other than the intervention of interest	Was follow up complete and if not, were differences between groups in terms of their follow up adequately described and analysed	Were participants analysed in the groups to which they were randomised	Were outcomes measured in the same way for treatment groups	Were outcomes measured in a reliable way	Was appropriate statistical analysis used	Was the trial design appropriate, and any deviations from the standard RCT design (individual randomisation, parallel groups) accounted for in the conduct and analysis of the trial
Ara et al. 2019 [28]	Yes	No	Yes	Yes	No	No	No	Not clear	Yes	Yes	Yes (except cognitive testing)	Yes	Yes
Aboud et al. 2011 [27]	Yes	No	Yes	Yes	No	No	No	Not clear	Yes	Yes	Not clear	Not clear	Yes
Vandana et al. 2014 [30]	Yes	No	Yes	Yes	Yes	No	No	Yes	Yes	Yes	yes	Yes	Yes

**Table 7:** Joanna Briggs Institute’s critical appraisal checklist of quasi-experimental studies for study quality assessment. [26] *Source**:* Joanna Briggs Institute’s critical appraisal checklist

Selected quasi experimental studies strengths and limitations (*n* = 3)									
Studies	Is it clear in the study what is the cause’ and what is the ‘effect’ (i.e., there is no confusion about which variable comes first)?	Were the participants included in any comparisons similar?	Were the participants included in any comparisons receiving similar treatment/care, other than the exposure or intervention of interest?	Was there a control group?	Were there multiple measurements of the outcome both pre- and post-intervention/exposure?	Was follow up complete and if not, were differences between groups in terms of their follow up adequately described and analysed?	Were the outcomes of participants included in any comparisons measured in the same way?	Were outcomes measured in a reliable way?	Was appropriate statistical analysis used?
Kushwaha KP et al. 2014 [29]	Yes	Yes	Yes	No	Yes	Yes	Yes	Yes	Yes
Singh et al. 2018 [31]	Yes	No	Yes	Yes	Yes	Yes	Yes	Yes	Yes
Abdullahi et al. 2019 [32]	Yes	No	No	Yes	Yes	Yes	Yes	Yes	Yes

**Table 8:** Summary of selected studies breastfeeding outcomes (*n* = 5)

Author and year	Breastfeeding outcomes
Ara et al. 2019 [28]	Colostrum feeding: PC + PCS group: 89.1%; 95% CI 83.5, 92.9, control group: 77.4%; 95% CI 69.5, 86.2
	Exclusive breastfeeding (at 5 months): 24 h recall -IG 92 (73.02), CG 34 (27.0)
	1 month recall -IG 81 (64.0), CG 25 (19.0)
Vandana et al. 2014 [30]	Early initiation of BF: IG: 67.5%, %, CG: 4.2%
	Extended BF:
	at 6–9 months- IG: A 88.2%, B1 86.0%, B2 88.0% and CG 87.5%
	at 9–12 months- IG: A 86.3%, B1 84.0%, B2 78.0% and CG 82.3%
Singh et al. 2018 [31]	Colostrum feeding: breastfeed within 1 h. among 6–11 months (C: -1.3, I: 9.5, *P* = 0.014)
Abdullahi et al. 2019 [32]	IYCF knowledge: changes on
	timely initiation of BF, I: 4.5(5.9), C: 3.7(4.1)
	exclusive BF: I: 43.8(6.6), C: 29.8(4.8)
Kushwaha et al. 2014 [29]	Early initiation of BF: T1 (I:71% vs. C:11%); adjOR:19.6 (95% CI 13.6, 28.2, *P* = ,0.0001), T2 (62% vs. 11%); adjOR:13.3 (95% CI 9.4, 18.9, *P* = ,0.0001)
	Exclusive breastfeeding: T1 (I: 50% vs. C: 7%); adjOR:13.6 (95% CI 7.6, 25.0, *P* = 0.0001) and T2 (I: 60% vs. C:7%); adjOR:20.5 (95% CI 11.3, 37.2, *P* = 0.0001)

*adjOR* Adjusted odds ratio, *BF* Breastfeeding, *BMI* Body mass index, *CG* Control group, *CI* Confidence interval, *IG* Intervention group, *IYCF* Infant and young child feeding, *SAM* Severe acute malnutrition

**Table 9:** Summary of selected studies of other outcomes (*n* = 5)

Author and year	Other significant reported outcomes
Ara et al. 2019 [28]	Child development (at 12): expressive communication IG: 0.0614, CG (− 0.0813), social–emotional activities IG 0.165, CG (− 0.219)
	Length & height increased in IG
Aboud et al. 2011 [27]	RFS Group:
	Home intervention (combined intervention (d = 0.38), for developmental outcome and hand washing with soap, responsive talk (d = 0.40), mouthful ingested (d = 0.35) and language skill (d = 0.35) was higher in intervention than in control group
	RFS + Group
Vandana et al. 2014 [30]	Self-feed, washing utensils before feeding, washing hands before feed, understand hunger cues: increased in intervention groups in comparison of control group
	Energy intake [27 h dietary recall]: Micronutrient intake was higher in intervention groups compare to control group
Singh et al. 2018 [31]	Maternal and child nutrition knowledge: fruits and vegetables are good for children 6–23·9 months (C: − 0·7, I: 10·6; *P* = 0·03) Maternal nutrition practices; maternal minimum dietary diversity (≥ 4 food groups; C: 3·6, I: 14·0; *P* = 0·03)
	Consumed two extra meals during lactation: among 6–11 months (C: -12.7, I: 0.9, *P* = 0.025)
	Took iron and folic acid during pregnancy: for at least 180 days (C:10.4, I: 20.9, *P* = 0.04)
	Antenatal care ≥ 4 times: among 6–11 months (C: 11.6, I: 18.4, *P* = 0.023)
	Postnatal care ≥ 3 times: among 6–11 months (C: 7.6, I: 9.9, *P* = 0.064)
Abdullahi et al 2019 [32]	IYCF knowledge: feeding up to 2 years; C: 30.3 (7.1), I: 31.1 (4.7),
	feeding during illness; C: 21.3 (7.3), I: 26.7 (5.2), responsive feeding; C: 14.6 (5.5), I: 31.7 (5.3)
	Change in BMI of caregivers; C: 2.119 (0.853), I: 3.361 (0.879)
	SAM among children; C: 2.9 (2.2), I: 1.1 (1.8)
	Nutritional status of children; C: 0.361 (0.158), I: 0.696 (0.665)

*adjOR* Adjusted odds ratio, *BF* Breastfeeding, *BMI* Body mass index, *CG* Control group, *CI* Confidence interval, *IG* Intervention group, *IYCF* Infant and young child feeding, *SAM* Severe acute malnutrition

## Abbreviations

BF: Breastfeeding
BMI: Body mass index
CG: Control group
CF: Complementary feeding
EBF: Exclusive breastfeeding
F2F: Father-to-father group
IG: Intervention group
IYCF: Infant and young child feeding
MDD: Minimum dietary diversity
MMF: Minimum meal frequency
MAD: Minimum acceptable diet
M2M: Mother-to-mother group
MSG: Mother support group
PC+PCS: Peer counselling plus psychological stimulation
SAM: Severe acute malnutrition
SD: Standard deviation
RCT: Randomised controlled trial
RFS: Responsive feeding and stimulation
RFS+: Responsive feeding and stimulation plus sprinkles
SES: Socio-economic status
WHO: World Health Organization
